# Olfactory memory in mild cognitive impairment and Alzheimer’s disease

**DOI:** 10.3389/fneur.2023.1165594

**Published:** 2023-06-02

**Authors:** Egle Audronyte, Vaiva Sutnikiene, Gyte Pakulaite-Kazliene, Gintaras Kaubrys

**Affiliations:** Clinic of Neurology and Neurosurgery, Faculty of Medicine, Institute of Clinical Medicine, Vilnius University, Vilnius, Lithuania

**Keywords:** Alzheimer’s disease, mild cognitive impairment – MCI, olfactory impairment, olfaction, olfactory memory

## Abstract

**Introduction:**

Olfaction is impaired in Alzheimer’s disease (AD). However, olfactory memory has rarely been examined. As the pathogenesis of AD remains largely unknown, collecting more data regarding the occurrence and progression of its symptoms would help gain more insight into the disease.

**Objective:**

To investigate olfactory memory and its relationship with verbal memory and other clinical features in patients with early-stage AD.

**Methods:**

Three groups of participants were enrolled in this study: patients with mild dementia due to AD (MD-AD, *N* = 30), patients with mild cognitive impairment due to AD (MCI-AD, *N* = 30), and cognitively normal older participants (CN, *N* = 30). All participants underwent cognitive evaluation (Clinical Dementia Rating scale, Mini Mental State Examination, Alzheimer’s Disease Assessment Scale–Cognitive Subscale, delayed verbal recall, and verbal fluency tests) and assessment of olfactory immediate and delayed recognition memory.

**Results:**

Olfactory immediate and delayed recognition memory scores were significantly lower in the MD-AD group than in the MCI-AD and CN groups. The MCI-AD and CN groups did not differ significantly [in both cases, Kruskal–Wallis test, *p* < 0.05; *post hoc* analysis revealed significant differences between the MD-AD and MCI-AD groups and between the MD-AD and CN groups (*p* < 0.05), and no significant difference between the MCI-AD and CN groups (*p* > 0.05)]. Verbal immediate recall, delayed recall after 5 min, and delayed recall after 30 min scores were significantly worse in the MD-AD and MCI-AD groups than in the CN group. MD-AD and MCI-AD groups did not differ significantly [in all cases Kruskal–Wallis test, *p* < 0.05; *post hoc* analysis revealed significant differences between MD-AD and CN groups, and MCI-AD and CN groups (*p* < 0.05) and no significant difference between MD-AD and MCI-AD groups (*p* > 0.05)]. Duration of AD symptoms was a strong predictor of both immediate and delayed olfactory recognition memory scores.

**Conclusion:**

Olfactory memory impairment was observed in patients with AD. The changes progress during the course of the disease. However, unlike verbal memory, olfactory memory is not significantly impaired in the prodromal stage of AD.

## 1. Introduction

The prevalence of Alzheimer’s disease (AD) and other dementias has increased by 160.84% in the 30 years from 1990 to 2019 and continues to increase (1). Consequently, the social and economic impacts of the disease are becoming major issues, making it a global healthcare priority (2, 3).

To achieve accurate diagnosis and identify effective treatment methods, a deeper understanding of the pathogenesis and progression of the disease is required. The amyloid β cascade hypothesis was proposed more than 30 years ago and has been continuously investigated ever since (4, 5). Another hallmark of AD is the hyperphosphorylation of tau proteins and formation of neurofibrillary tangles (6). However, AD is a complex condition that cannot be explained exclusively by these mechanisms. Many other processes are proposed as contributing to AD, especially neuroinflammation and mitochondrial dysfunction (6, 7). Nevertheless, despite extensive research on the subject, the pathogenesis of AD remains largely unknown. An imbalance of various neurotransmitters is observed in patients with AD, with cholinergic deficit being the most recognized feature (7). As cholinergic pathways are involved in various processes, including memory and olfactory information processing, a deeper understanding of AD symptoms and their progression would help gain more insight into the processes of the disease.

Olfactory impairment has recently gained attention as a common and early sign of AD that precedes cognitive decline by several years (8–10). However, most studies have focused on odor identification testing, and other olfactory functions have rarely been analyzed. Especially sparse are data regarding olfactory memory.

Animal studies have yielded promising results. Olfactory memory deficits have been observed in mouse models of AD (11, 12). Olfactory memory impairment and altered functioning of olfactory network was also found in apolipoprotein E ɛ4 (ApoE4) knock-in mice (13). Studies on cognitively unimpaired human subjects at higher risk of AD (ApoE4 carriers) have confirmed these findings. ApoE4 carriers have impaired olfactory memory abilities (14, 15) and altered activation on functional magnetic resonance imaging (fMRI) during olfactory memory tasks (16). In healthy elderly subjects reduced olfactory memory abilities were found to be associated with deficits in executive functioning (17).

However, few studies have been conducted in patients with AD. Furthermore, the results are inconsistent, with some authors finding olfactory memory to be affected in patients with AD (18, 19), while others found only odor identification to be impaired, with no deficits in olfactory memory (20). The lack of research on olfactory memory probably stems from the difficulties in assessing this function. Typically, odor familiarity ratings or various odor recognition tasks are employed for this purpose (18, 21, 22). However, there are no universally accepted methods for assessing olfactory memory, with some authors even measuring the verbal recall of previously presented odors (23).

The objective of our study was to address this knowledge gap and investigate olfactory memory function in patients with early stage AD. We further aimed to investigate the relationship between olfactory and verbal memory, as well as other clinical features. Since the anatomical structures involved in olfactory and verbal memory processes differ, collecting more data regarding specific patterns of olfactory and verbal memory impairment in patients with AD would help gain more insight into the progression of AD.

## 2. Materials and methods

### 2.1. Participants

Ninety participants were enrolled in the study: 30 cognitively normal older participants (CN), 30 with mild cognitive impairment due to Alzheimer’s disease (MCI-AD), and 30 with mild dementia due to AD (MD-AD).

AD was diagnosed according to the National Institute on Aging-Alzheimer’s Association (NIA/AA) criteria for probable AD (24). MCI-AD was diagnosed according to the NIA/AA criteria for MCI due to AD (25). The cognitively healthy older participants had no cognitive complaints or neurological disorders.

MD-AD patients had a Clinical Dementia Rating (CDR) total score of 1, MCI-AD patients had a CDR total score of 0.5, and CN participants had a CDR total score of 0.

The participants with MCI-AD and with MD-AD were recruited form the Memory Clinic in Vilnius University Hospital Santaros Klinikos. Cognitively normal older participants were recruited form the primary care clinic in the same hospital.

Patients were only enrolled in the study if they were treatment-naïve or were taking a stable dose of an acetylcholinesterase inhibitor (AChEI) for at least 3 months.

Participants were excluded from the study if they had: other central nervous system disorders; a Hachinski Ischemic Score ≥ 4, indicating possible significant cerebrovascular disease; or previous significant head trauma. Participants with psychiatric conditions such as psychosis, substance abuse, significant depression (Geriatric Depression Scale score > 9), and those taking psychoactive medications were also excluded. Participants with conditions potentially affecting olfaction were also excluded from the study (smoking, nasal trauma or surgery, significant exposure to volatile substances, and recent viral infections).

This study was approved by the Vilnius Regional Bioethics Committee (Approval Number 2021/6–1,355-830). All participants agreed to participate in the study, were informed of the study procedures, and provided written informed consent by signing relevant written informed consent forms.

### 2.2. Assessments of cognitive function

The Mini Mental State Examination (MMSE) was performed to evaluate global cognition.

For more detailed evaluation, the Alzheimer’s Disease Assessment Scale–Cognitive Subscale (ADAS-Cog) was administered. Additionally, delayed recall was evaluated after 5 min and after 30 min.

The verbal fluency score (VFS), comprising phonemic verbal fluency (PAS) and categorical verbal fluency (animals), was also tested.

Severity of symptoms was quantified using the CDR scale.

### 2.3. Assessment of olfactory memory

The olfactory memory assessment task was designed using odors from the standard validated Sniffin’ Sticks odor identification test (Burghart, Wedel, Germany).

The Sniffin’ Sticks odor identification test consists of 16 odors presented in felt-tip pens.

In the encoding phase of the olfactory memory task, five odors were randomly assigned to each participant (target odors). Each of these five odors was presented for 3 s with the tip of the pen placed approximately 2 cm in front of both nostrils. The participants were instructed to memorize the odors without verbal clues.

Immediate olfactory recognition memory was assessed immediately after the encoding phase. Five new odors were randomly assigned to each participant (distractors). Distractors were presented with the target odors in a randomized manner. Each of the 10 odors was presented for 3 s with the tip of the pen placed approximately 2 cm in front of both nostrils. Participants were instructed to choose whether the odor was new or presented previously (target odor). The immediate odor recognition score was the number of correct answers (0–10).

Delayed olfactory recognition memory was tested 30 min after the encoding phase. Five new odors were randomly assigned to each participant (second group of distractors). Distractors were presented with the target odors in a randomized manner. Each of the 10 odors was presented for 3 s with the tip of the pen placed approximately 2 cm in front of both nostrils. Participants were instructed to choose whether the odor was new or presented previously (target odor). The delayed odor recognition score was the number of correct answers (0–10).

A time interval of 30 s was kept between odors.

All participants were instructed not to drink or eat anything for at least 15 min prior to testing. The examiner wore odorless gloves and the participants wore a blindfold.

### 2.4. Data analysis

Statistical analysis was performed with IBM SPSS Statistics version 26.0 (IBM Corp., Armonk, NY, United States).

The normality of the data distribution was tested using the Shapiro–Wilk test. Differences between groups were analyzed using a two-tailed chi-square test (categorical variables), Mann–Whitney U test (numerical variables, comparison between two groups), and Kruskal–Wallis test (numerical variables, comparison between three groups).

The Spearman rank correlation coefficient was used to determine correlation between variables.

Linear regression models were created to analyze the predictions of continuous variables.

A value of *p* of <0.05 was considered statistically significant.

## 3. Results

### 3.1. Demographic and clinical characteristics

Cognitively normal older participants (CN), patients with mild cognitive impairment due to Alzheimer’s disease (MCI-AD), and patients with mild dementia due to Alzheimer’s disease (MD-AD) did not differ according to sex (two-tailed chi-square test, *p* > 0.05). There were also no differences according to education, Geriatric Depression Scale (GDS) results, or Hachinski Ischemic Score (HIS) (Kruskal–Wallis test, *p* > 0.05).

The CN and MCI-AD groups did not differ in age. The median age of the MD-AD group was significantly higher (Kruskal–Wallis *p* < 0.05; *post hoc* analysis revealed no significant difference between the CN and MCI-AD groups, and significant differences between the CN and MD-AD, and MCI-AD and MD-AD groups; Cohen’s *d* = 0.906).

As expected, patients with MD-AD had a significantly longer duration of AD symptoms than those with MCI-AD (Mann–Whitney *U* test, *p* < 0.001; Cohen’s *d* = 1.132). There were significantly more patients taking AChEIs in the MD-AD group than in the MCI-AD group (two-tailed chi-square test, *p* < 0.05).

The demographic and clinical characteristics of the participants are presented in Table 1.

**Table 1:** Demographic and clinical characteristics of the participants.

	CN (*N* = 30)	MCI-AD (*N* = 30)	MD-AD (*N* = 30)
Male, *n* (%)*	13 (43.33%)	13 (43.33%)	12 (40%)
Age (years)**	74 (68.75–76)	72 (67.75–77.25)	78 (75–79.25)
Years of education*	15 (13.5–16)	16 (14–16)	16 (13–16)
HIS*	1 (0–1)	1 (0–1)	1 (1–1.25)
GDS*	5.5 (4–6.25)	5.5 (4–6)	5 (4–6.25)
Duration of AD symptoms (in years)***	N/A	3 (2–3)	4 (3–5)
Use of AChEI, *n* (%)***	N/A	3 (10%)	14 (46.7%)

CN, cognitively normal; MCI-AD, mild cognitive impairment due to Alzheimer’s disease; MD-AD, mild dementia due to Alzheimer’s disease; HIS, Hachinski Ischemic Score; GDS, geriatric depression scale; AChEI, acetylcholinesterase inhibitor. Data are presented as median and interquartile range unless otherwise specified.

* Groups do not differ significantly.

** MD-AD group differs significantly from CN and MCI groups. CN and MCI groups do not differ significantly from each other.

*** MD-AD group differs significantly from MCI-AD group.

### 3.2. Cognitive characteristics

The three groups differed significantly in cognitive assessment tasks (CDR sum of boxes, MMSE, ADAS-Cog, and combined VFS) (in all cases, Kruskal–Wallis test *p* < 0.05, and *post hoc* analysis revealed significant differences between all three groups).

Results of cognitive tests are presented in Table 2.

**Table 2:** Cognitive assessment of the participants.

	CN (*N* = 30)	MCI-AD (*N* = 30)	MD-AD (*N* = 30)
CDR sum of boxes*	0 (0–0)	2 (1.5–2.5)	5 (4.5–5.5)
MMSE*	29 (29–30)	26 (25–26)	22 (21–23)
ADAS-Cog*	5.33 (4.59–7)	11.33 (9.17–13.75)	17.67 (15.17–20.33)
VFS*	57.5 (43–63)	41 (35–50.75)	29.5 (21–39)

CN, cognitively normal; MCI-AD, mild cognitive impairment due to Alzheimer’s disease; MD-AD, mild dementia due to Alzheimer’s disease; CDR, clinical dementia rating; MMSE, mini mental state examination; ADAS-Cog, Alzheimer’s disease assessment scale–cognitive subscale; VFS, combined verbal fluency score.

Data are presented as median and interquartile range.

* All three groups differ significantly.

Verbal memory was also analyzed. Immediate recall (third trial on the ADAS-Cog word recall task), delayed recall after 5 min, and delayed recall after 30 min all differed significantly between the CN and MCI-AD groups, and the CN and MD-AD groups, but did not differ significantly between the MCI-AD and MD-AD groups [in all three cases, Kruskal–Wallis test *p* < 0.05; *post hoc* analysis revealed significant differences between the CN and MCI-AD groups, and CN and MD-AD groups (*p* < 0.05), and no significant difference between the MCI-AD and MD-AD groups (*p* > 0.05); Cohen’s *d* = 2.518, 2.992 and 3.108, respectively].

**Figure 1:**
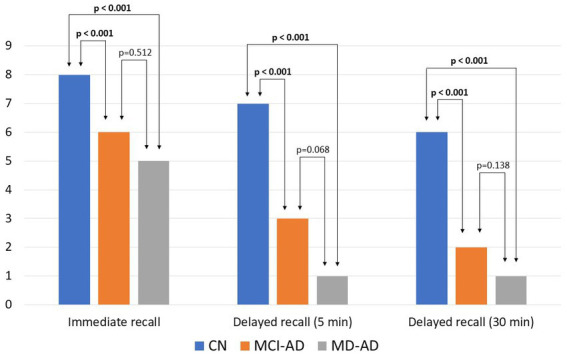
Results of verbal recall memory tasks in three groups of participants. Bars represent medians. Kruskal–Wallis test found the three groups to differ significantly (*p* < 0.05). *Post hoc* analysis revealed significant differences between the CN and MCI-AD groups, and CN and MD-AD groups (*p* < 0.05), and no significant difference between the MCI-AD and MD-AD groups (*p* > 0.05). CN, cognitively normal; MCI-AD, mild cognitive impairment due to Alzheimer’s disease; MD-AD, mild dementia due to Alzheimer’s disease.

The results [median and interquartile range (IQR)] of immediate recall in the CN, MCI-AD, and MD-AD groups were 8 (8–9), 6 (5–7), and 5 (4–6), respectively. Results of delayed recall after 5 min were 7 (6–7), 3 (1–4.25), and 1 (0–2), respectively. Results of delayed recall after 30 min were 6 (6–7), 2 (1–3.25), and 1 (0–1.25), respectively. The results of the verbal recall memory task are shown in Figure 1.

The results of the verbal memory tasks were compared between patients with AD (MCI-AD and MD-AD participants) taking AChEIs and treatment-naïve patients.

Patients on AChEIs did not differ from treatment-naïve patients in immediate recall [median and IQR 5 (4.5–6.5) and 6 (4–6), respectively; Mann–Whitney *U* test, *p* = 0.762], delayed recall after 5 min [median and IQR 2 (1–2.5) and 2 (1–4), respectively; Mann–Whitney *U* test, *p* = 0.617], or delayed recall after 30 min [median and IQR 1 (0–2) and 1 (0–3), respectively; Mann–Whitney *U* test, *p* = 0.852] tasks. However, the duration of AD symptoms was significantly longer in patients taking AChEIs than in those who were not [median and IQR 4 (3.5–5) and 3 (2–3), respectively; Mann–Whitney *U* test, *p* < 0.001; Cohen’s *d* = 1.323].

Upon analyzing the separate groups, the results remained the same. In the MCI-AD group there were no significant differences between patients on AChEIs and patients not taking them in immediate recall [median and IQR 6 (5–6) and 7 (5–7), respectively; Mann–Whitney *U* test, *p* = 0.283], delayed recall after 5 min [median and IQR range 4 (1–4) and 3 (1–4), respectively; Mann–Whitney *U* test, *p* = 0.554], and delayed recall after 30 min [median and IQR 3 (0–3) and 2 (1–3), respectively; Mann–Whitney *U* test, *p* = 0.600].

In the MD-AD group there were also no significant differences between patients on AChEIs and patients not taking them in immediate recall [median and IQR 5 (4–6) and 4.5 (3.25–6), respectively; Mann–Whitney U test, *p* = 0.400], delayed recall after 5 min [median and IQR 2 (0.75–2) and 1 (0–2.75), respectively; Mann–Whitney *U* test, *p* = 0.423], and delayed recall after 30 min [median and IQR 1 (0–2) and 0 (0–1), respectively; Mann–Whitney *U* test, *p* = 0.179].

The difference in the duration of AD symptoms between patients on treatment and untreated participants remained significant both in the MCI-AD [median and IQR 4 (3–4) and 2 (2–3), respectively; Mann–Whitney *U* test, *p* = 0.026; Cohen’s *d* = 0.85], and in the MD-AD groups [median and IQR 6 (5–6) and 3 (3–4), respectively; Mann–Whitney *U* test, *p* = 0.01; Cohen’s *d* = 1.045].

### 3.3. Olfactory memory characteristics

Olfactory immediate recognition memory and olfactory delayed recognition memory were significantly worse in patients with MD-AD than in those with MCI-AD or CN. The MCI-AD and CN groups did not differ significantly from each other [in both cases, Kruskal–Wallis test, *p* < 0.05; *post hoc* analysis revealed significant differences between the MD-AD and MCI-AD groups and the MD-AD and CN groups (*p* < 0.05), and no significant difference between the MCI-AD and CN groups (*p* > 0.05); Cohen’s *d* = 0.966 and 0.852, respectively].

**Figure 2:**
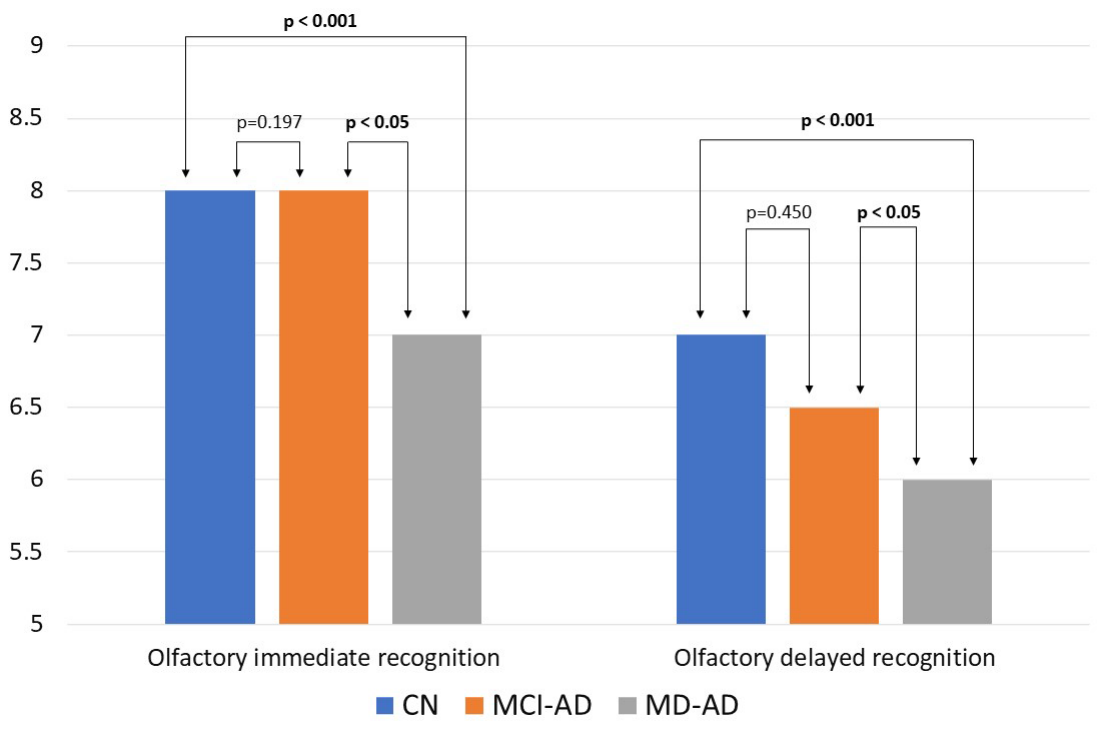
Results of olfactory recognition memory tasks in three groups of participants. Bars represent medians. Kruskal–Wallis test found the three groups to differ significantly (*p* < 0.05). *Post hoc* analysis revealed significant differences between the MD-AD and MCI-AD groups and the MD-AD and CN groups (*p* < 0.05), and no significant difference between the MCI-AD and CN groups (*p* > 0.05). CN, cognitively normal; MCI-AD, mild cognitive impairment due to Alzheimer’s disease; MD-AD, mild dementia due to Alzheimer’s disease.

The median and IQR of immediate recognition memory in the MD-AD, MCI-AD, and CN groups were 7 (6–7), 8 (6–8.25), and 8 (7–9), respectively. Results of delayed recognition memory were 6 (5–6.25), 6.5 (5.75–8), and 7 (6–8), respectively. The results of the olfactory recognition memory tasks are shown in Figure 2.

In patients with AD (MCI-AD and MD-AD groups), olfactory immediate recognition memory scores were significantly correlated with the duration of AD symptoms (Spearman rho = −0.366, *p* < 0.05), CDR sum of boxes (Spearman rho = −0.328, *p* < 0.05), and VFS (Spearman rho = 0.355, *p* < 0.05). Olfactory delayed recognition memory scores correlated significantly with the duration of AD symptoms (Spearman rho = −0.360, *p* < 0.05), CDR sum of boxes (Spearman rho = −0.317, *p* < 0.05), VFS (Spearman rho = 0.303, *p* < 0.05), and delayed verbal recall after 5 min (Spearman rho = 0.258, p < 0.05). Neither olfactory immediate recognition memory scores, nor olfactory delayed recognition memory scores correlated significantly with age (Spearman rho = −0.194, *p* = 0.138 and Spearman rho = −0.226, *p* = 0.082, respectively).

Multiple linear regression models were created to determine which variables that correlated with olfactory memory scores in AD patients could significantly predict them. Additionally, age, sex, and GDS scores were included in the models as factors known to influence memory and olfaction in the general population.

In the model with age, sex, GDS score, duration of AD symptoms, CDR sum of boxes, and VFS as independent variables and olfactory immediate recognition as the dependent variable, the overall regression was significant (*R*^2^ = 0.247, *F* = 2.904, *p* = 0.016). The strongest predictor was duration of AD symptoms [*β* = −0.261, *B* = −0.288, 95% Confidence interval (95% CI) (−0.597, 0.02); *p* = 0.066]. In a stepwise regression model, duration of AD symptoms [*β* = −0.290, *B* = −0.321, 95% CI (−0.59, −0.051); *p* = 0.021] and VFS [*β* = 0.306, *B* = 0.033, 95% CI (0.007, 0.06); *p* = 0.015] remained the only significant predictors of olfactory immediate recognition memory score.

In the model with age, sex, GDS score, duration of AD symptoms, CDR sum of boxes, VFS, and delayed verbal recall after 5 min as independent variables and olfactory delayed recognition as dependent variable, the overall regression was also significant (*R*^2^ = 0.232, *F* = 2.249, *p* = 0.045). The strongest predictor was duration of AD symptoms as well [*β* = −0.302, *B* = −0.309, 95% CI (−0.609, −0.010); *p* = 0.043]. In a stepwise regression model duration of AD symptoms [*β* = −0.291, *B* = −0.299, 95% CI (−0.553, −0.044); *p* = 0.022] and VFS [*β* = 0.268, *B* = 0.027, 95% CI (0.002, 0.052); *p* = 0.035] remained the only significant predictors of olfactory delayed recognition memory score.

The results of the olfactory memory tasks were compared between patients with AD (MCI-AD and MD-AD participants) taking AChEIs and treatment-naïve patients. Patients on AChEIs had significantly worse results than treatment-naïve patients in both olfactory immediate recognition [median and IQR 6 (6–7) and 7 (6–8), respectively; Mann–Whitney *U* test, *p* = 0.024; Cohen’s *d* = 0.592] and olfactory delayed recognition [median and IQR 6 (6–6) and 6 (5–6), respectively; Mann–Whitney *U* test, *p* = 0.025; Cohen’s *d* = 0.585] tasks.

Analysis of the separate groups revealed that these differences were no longer significant at the group level. In the MCI-AD group, there were no significant differences between patients on AChEIs and patients not taking them, neither in olfactory immediate recognition [median and IQR 6 (6–6) and 8 (6–8) respectively; Mann–Whitney *U* test, *p* = 0.600], nor in olfactory delayed recognition [median and IQR 6 (5–6) and 7 (6–8), respectively; Mann–Whitney *U* test, *p* = 0.387].

In the MD-AD group, there were also no significant differences between patients on AChEIs and those not taking them, neither in olfactory immediate recognition [median and IQR 6 (5.75–7) and 7 (6–7), respectively; Mann–Whitney *U* test, *p* = 0.208], nor in olfactory delayed recognition [median and IQR 5.5 (5–6) and 6 (5–7), respectively; Mann–Whitney *U* test, *p* = 0.313].

## 4. Discussion

We found olfactory recognition memory to be impaired in AD patients in the current study. This confirms the findings of other authors who also found olfactory memory to be worsened in AD (18, 19). Although not all studies in patients with AD confirm this finding (20), animal studies suggest that impairment of olfactory memory is indeed a feature of AD (11, 12).

Data on patients with early-stage AD are limited. In a study performed in 2008, authors found no olfactory memory deficits in prodromal AD (MCI patients) (20). However, in a more recent work, researchers revealed olfactory memory to be impaired in MCI, as well as in patients with subjective cognitive decline (SCD) (18). In the current study, we found olfactory recognition memory (immediate, as well as delayed) to be impaired in patients with mild dementia, however, performance of MCI-AD participants did not differ significantly from cognitively normal participants.

Olfactory recognition memory (immediate, as well as delayed) scores correlated with the duration of the symptoms in AD patients. Furthermore, multiple linear regression analysis showed that duration of AD symptoms was a strong predictor of olfactory recognition memory (immediate, as well as delayed) scores. Thus, we can conclude that olfactory memory is a symptom of AD and that deficits in olfactory memory progress during the course of the disease. However, these changes did not reach the level of statistical significance during the prodromal stage of the disease (MCI due to AD).

However, verbal memory testing yielded different results. Verbal recall memory (immediate, as well as delayed) was already significantly impaired in MCI-AD patients. However, the patients with MD-AD did not differ significantly from those with MCI-AD. This is not surprising as episodic verbal memory impairment is an early and prominent symptom of AD, with subtle changes occurring even at the preclinical stage (26). In this study, verbal memory deficits were highly pronounced in patients with MCI-AD. As a result, even though patients with mild dementia tended to have worse results than patients with MCI, especially in the delayed recall task, these differences did not reach statistical significance. In addition, the ADAS-Cog recall task may lack sensitivity for differentiating between patients with MCI and mild dementia, and more complex tasks are needed for this purpose. This is consistent with the results from previous studies, where the addition of a delayed recall task to the ADAS-Cog also increased the accuracy of testing MCI participants but did not improve the accuracy of testing AD patients with dementia (27).

Even today, the specific brain regions responsible for human olfactory memory are poorly understood; however, multiple structures are known to be involved in this process, and this network differs significantly from verbal memory (28–30). The results of our study also highlighted the differences between these two types of memory, as their impairment occurs at different stages of the disease.

In the current study, treatment-naïve patients showed better olfactory memory results than patients taking AChEIs. However, the duration of AD symptoms was significantly shorter in treatment-naive patients. Longer disease duration accounts for the more pronounced olfactory memory impairment in patients taking AChEIs. It is interesting to note that verbal memory did not differ significantly between patients taking AChEIs and those who were not, despite the longer duration of the disease. Thus, we can conclude that AChEI treatment had a significant effect on verbal memory and was able to compensate for the longer progression of the disease; however, the same effect was not observed for olfactory memory. Thus, not only do olfactory and verbal memory deficits manifest in different patterns in patients with AD, but the response to cholinergic stimulation is also distinct.

In animal studies, cholinergic activation has been found to improve olfactory dysfunction (31–33). However, results regarding the impact of AChEIs on olfactory function in patients with AD are inconsistent. Some studies have found that olfactory function is improved by treatment with AChEIs (34, 35) and have even suggested that atropine challenge is indicative of a cognitive response to AChEI treatment; however, further studies did not confirm these findings (36, 37). It is important to note that odor identification was tested in these studies, and olfactory memory was not specifically analyzed. Thus, the different effects of AChEIs on olfactory identification and memory could not be excluded. In the current study, there was a significant difference in olfactory memory scores depending on AChEI status in the sample of all patients with AD. However, similar differences were not found in the MCI-AD and MD-AD groups separately, even though the difference in disease duration remained significant. Therefore, a positive effect of cholinergic stimulation on olfactory memory cannot be excluded, although it is not as substantial as its effect on verbal memory.

This study has a few limitations. First, the cross-sectional design limited the accuracy of the conclusions regarding longitudinal changes during the course of AD. Second, biomarkers of amyloid deposition and neuronal degeneration were not tested, thus preventing the analysis of their relationship with the findings. Finally, in order to confirm these results, further research with larger samples of participants is needed.

In conclusion, our findings indicated that olfactory memory is impaired in patients with AD. These deficits progress over the course of the disease. However, unlike verbal memory, olfactory memory is not significantly impaired in the prodromal stage of AD.

## Data availability statement

The raw data supporting the conclusions of this article will be made available by the authors, without undue reservation.

## Ethics statement

The studies involving human participants were reviewed and approved by the Vilnius Regional Bioethics Committee. The patients/participants provided their written informed consent to participate in this study.

## Author contributions

EA: study design and concept, data collection and analysis, and manuscript editing. VS and GP-K: data collection, data analysis, and manuscript editing. GK: study design and concept, data analysis, and manuscript editing. All authors contributed to this article and approved the final manuscript.

## Conflict of interest

The authors declare that the research was conducted in the absence of any commercial or financial relationships that could be construed as a potential conflict of interest.

## Publisher’s note

All claims expressed in this article are solely those of the authors and do not necessarily represent those of their affiliated organizations, or those of the publisher, the editors and the reviewers. Any product that may be evaluated in this article, or claim that may be made by its manufacturer, is not guaranteed or endorsed by the publisher.
